# Supplementary material for: Thermodynamics of the Ising Model Encoded in Restricted Boltzmann Machines
Source: Entropy (Basel). 2022 Nov 22;24(12):1701. doi: 10.3390/e24121701 (PMC9777808; doi:10.3390/e24121701)
Supplement: Supplementary file 1 [file entropy-24-01701-s001.zip › entropy-2008042-supplementary.pdf]

Supplementary Materials to Thermodynamics of the Ising model encoded in restricted Boltzmann machines

Jing Gu[1] and Kai Zhang[1,2,*]

[1]*Division of Natural and Applied Sciences, Duke Kunshan University, Kunshan, Jiangsu, 215300, China*

[2]*Data Science Research Center (DSRC), Duke Kunshan University, Kunshan, Jiangsu, 215300, China*

This supplementary document contains detailed results about the training of RBMs, the constrained RBM with biases set to zero, variance of filter sum, correlation of inverse filter, and reconstructed thermal quantities by RBMs. More examples of filters of single temperature RBMs at different temperatures and of the all temperature RBM are also included.

I. TRAINING OF RBMS

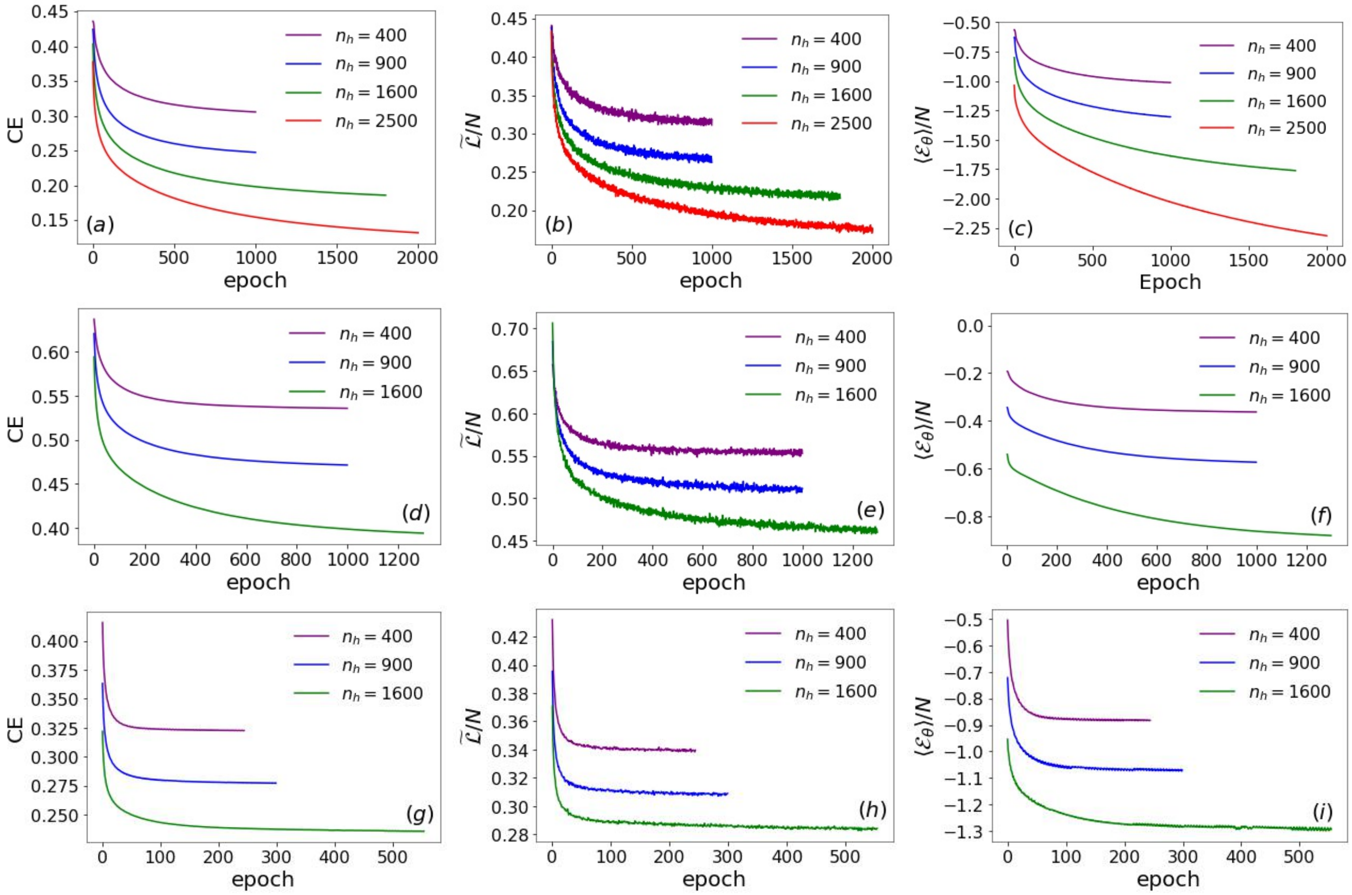

Figure S1. Evolution of cross entropy CE, pseudo-likelihood $\widetilde{\mathcal{L}}$, and mean visible energy $\langle \mathcal{E}_{\boldsymbol{\theta}} \rangle$ during the training of RBMs with different number n_h of hidden units for (a-c) T-RBM of the $2d$ Ising model at $T = 2.25$, (d-f)T-RBM of the $3d$ Ising model at $T = 4.5$, and (g-i) $\cup T$-RBM of the $2d$ Ising model.

* kai.zhang@dukekunshan.edu.cn

II. CONSTRAINED RBM

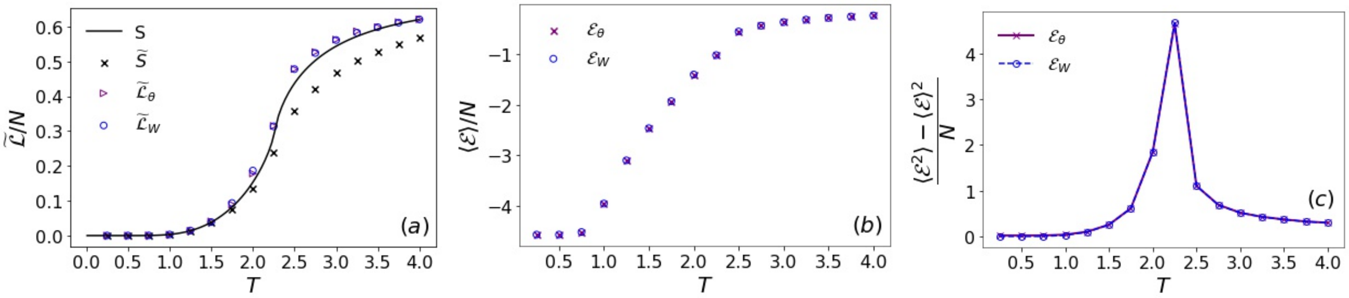

Figure S2. (a) Pseudo-likelihood $\widetilde{\mathcal{L}}$ per spin, (b) mean, and (c) variance of visible energy $\mathcal{E}$ of T-RBM ($\boldsymbol{\theta}$) and constrained T-RBM ($\mathbf{W}$) with biases set to zero for the $2d$ Ising model. $n_h = 400$. Entropy S and pseudo-entropy $\widetilde{S}$ are also shown in (a).

III. VARIANCE OF FILTER SUM AND CORRELATION OF INVERSE FILTER

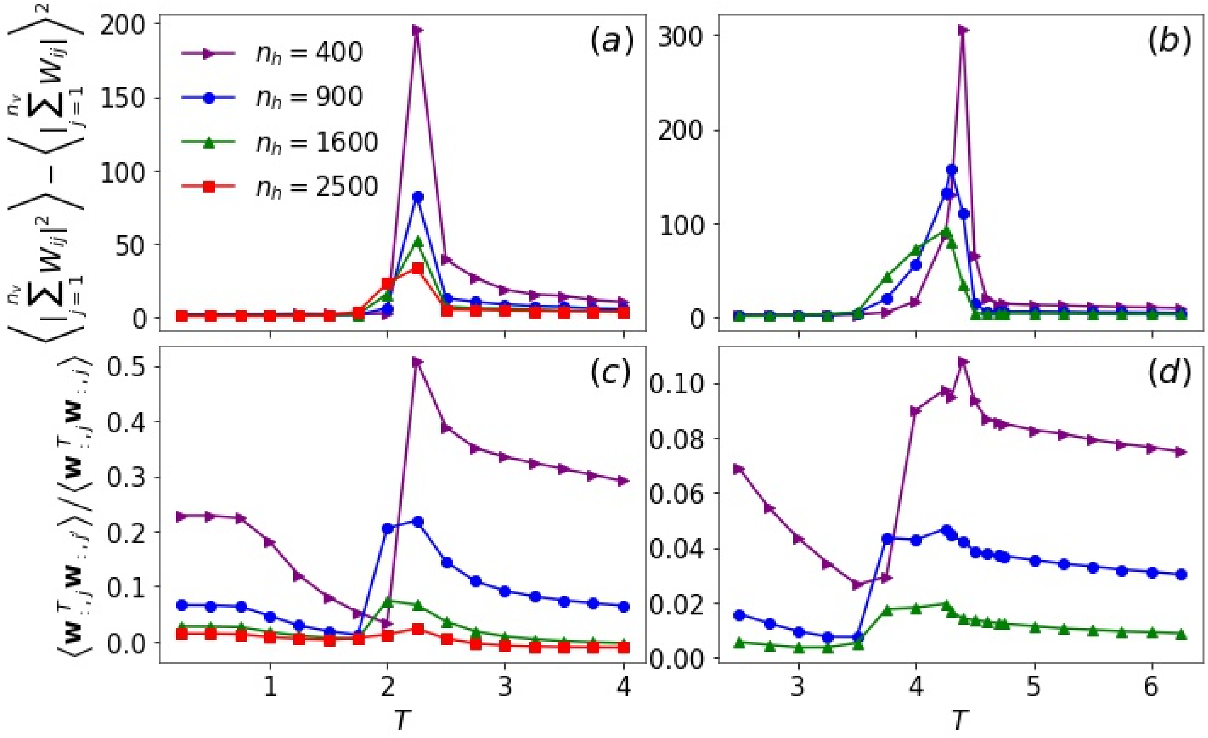

Figure S3. (a-b) Variance $\left\langle \left| \sum_{j=1}^{n_v} W_{ij} \right|^2 \right\rangle - \left\langle \left| \sum_{j=1}^{n_v} W_{ij} \right| \right\rangle^2$ of the filter sum and (c-d) correlation between a pair of inverse filters $\mathbf{w}_{:,j}$ and $\mathbf{w}_{:,j'}$ (normalized by auto-correlation) with spin-spin distance $r_{jj'} = 3$ as a function of temperature for $2d$ (a,c) and $3d$ (b,d) Ising models captured by T-RBMs.

IV. RECONSTRUCTION OF RBMS

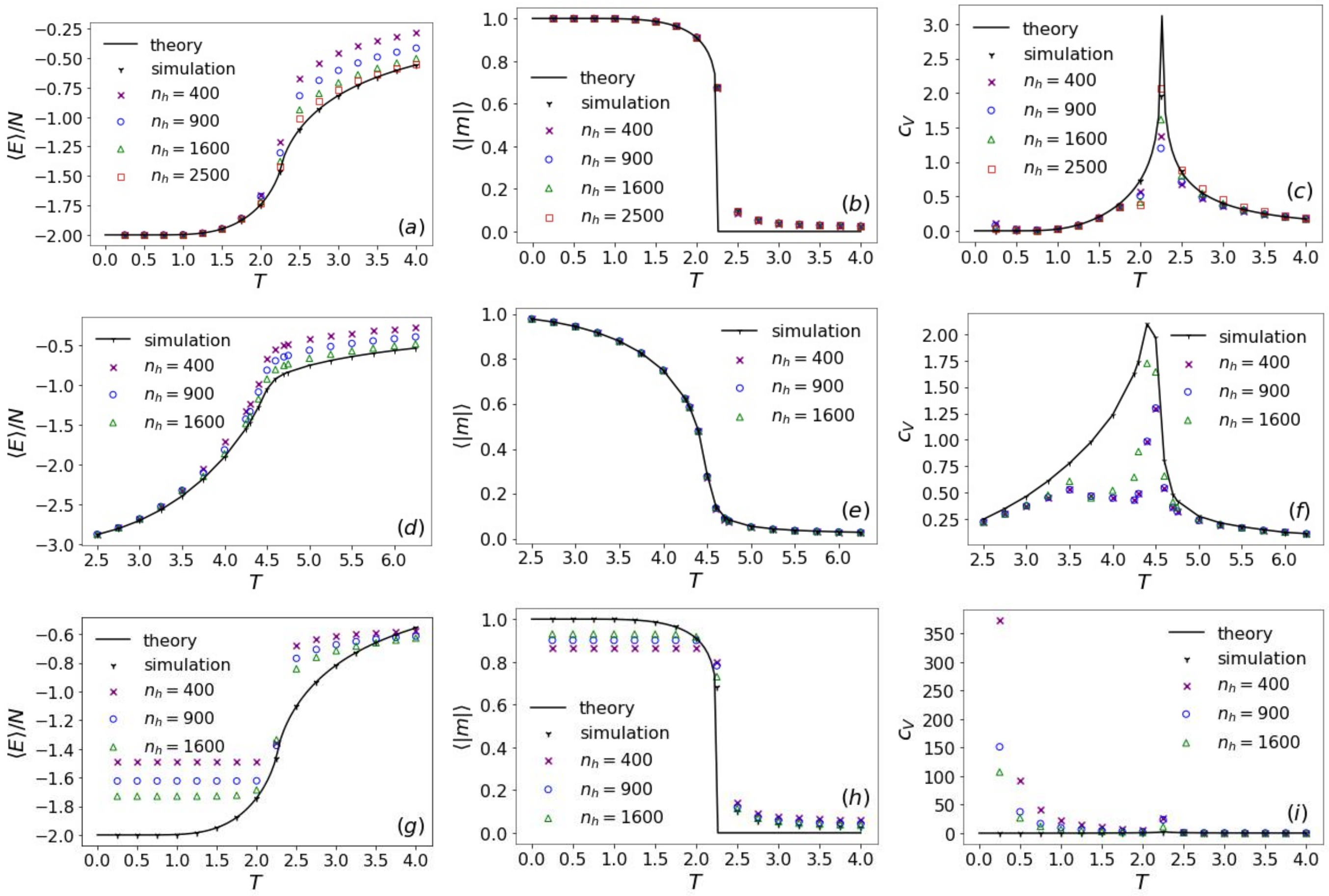

Figure S4. (a,d,g) Internal energy, (b,e,h) magnetization, and (c,f,i) specific heat of $2d$ (a-c,g-i) and $3d$ (d-f) Ising configurations reconstructed by T-RBMs (a-f) and the $\cup T$-RBM (g-i) with different number n_h of hidden units in comparison with theoretical and Monte Carlo simulation values.

V. FILTERS OF RBMS

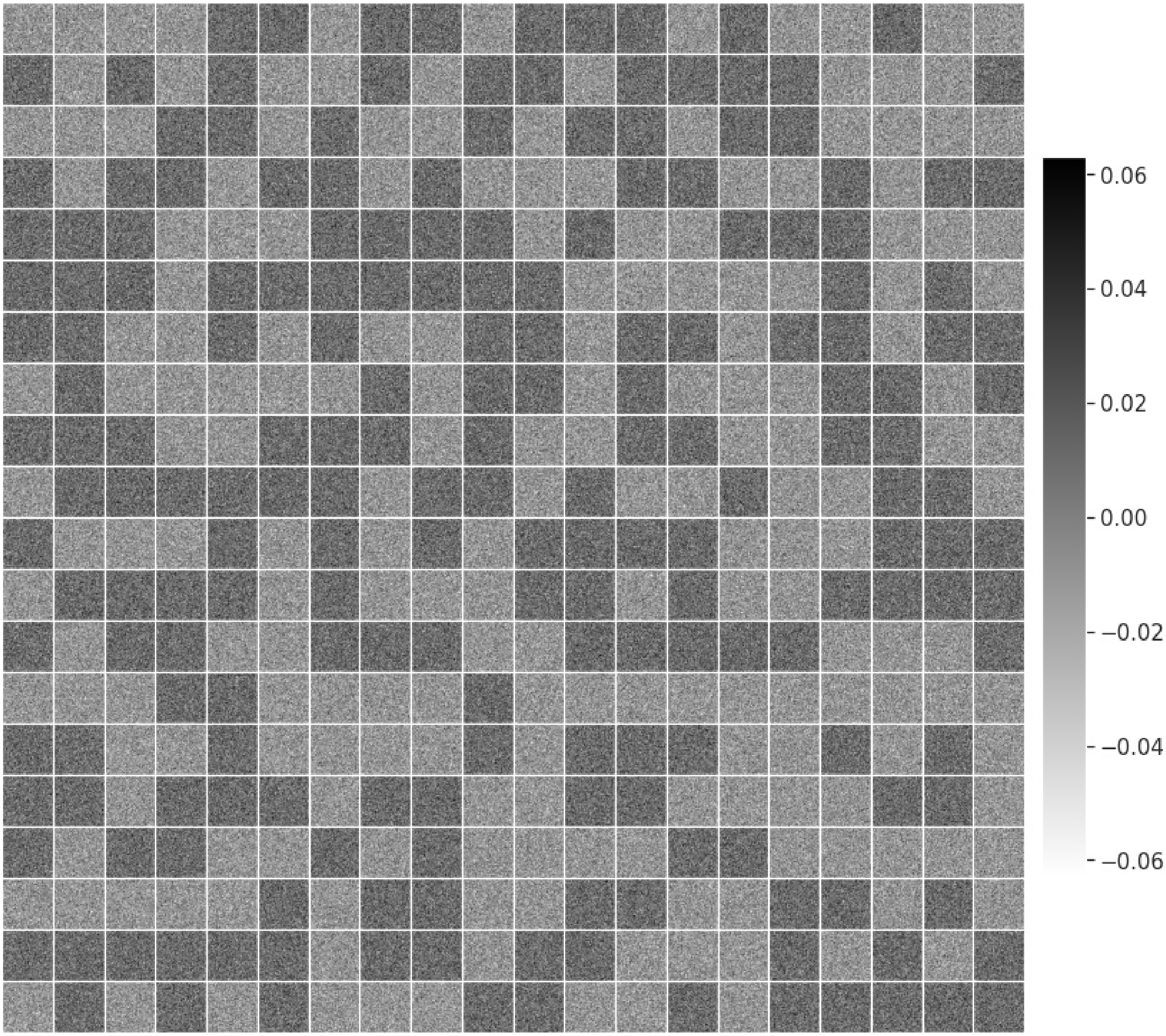

Figure S5. Filters $\mathbf{w}_i^T$ of T-RBMs with $n_h = 400$ for the $2d$ Ising model at temperature $T = 1.0$. Mean of standard deviation $\langle std_{T=1.0}\rangle$ of $\mathbf{w}_i^T$ is calculated. The colorbar range is set within $[-3 \times \langle std_{T=1.0}\rangle, 3 \times \langle std_{T=1.0}\rangle]$.

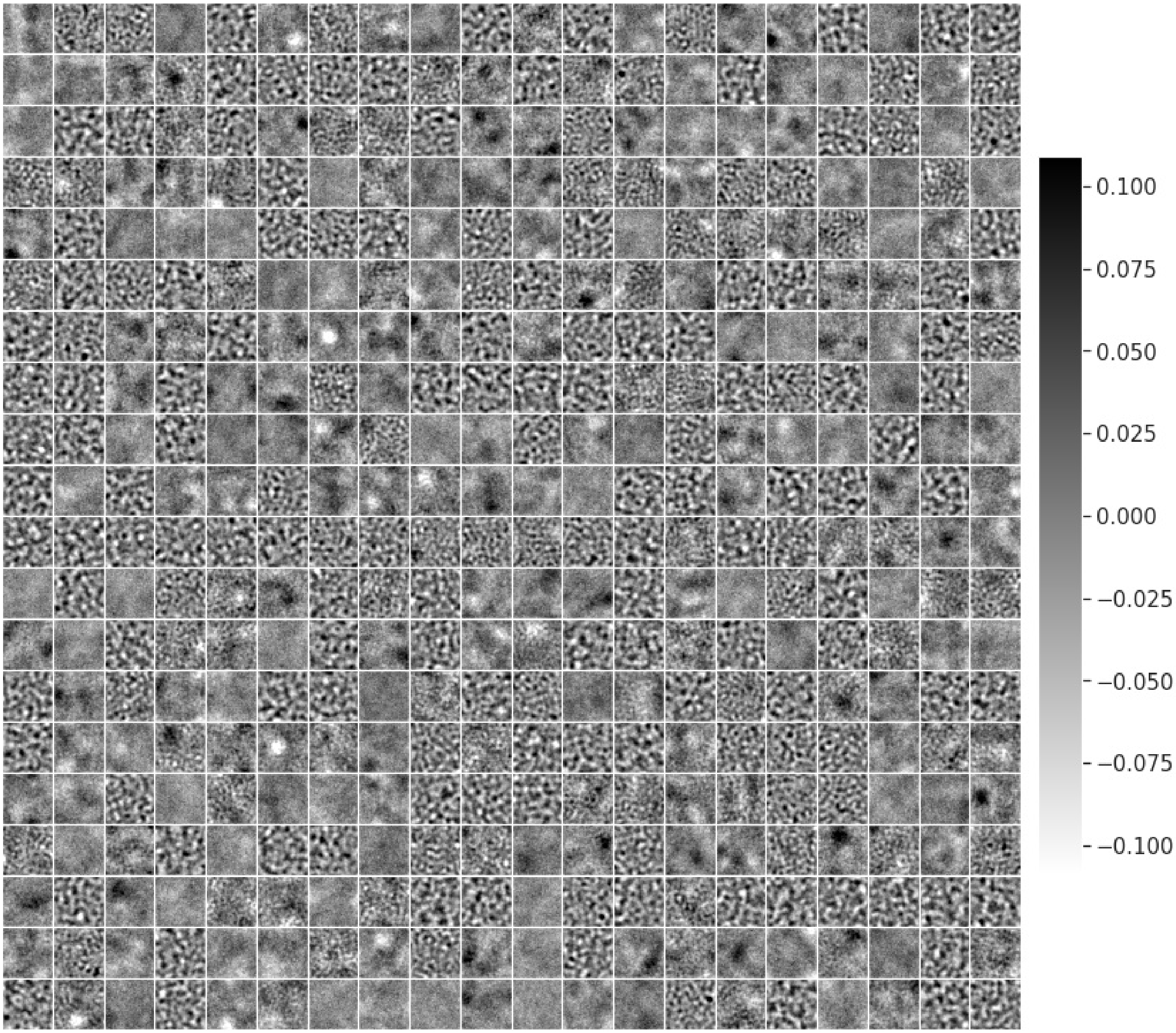

Figure S6. Filters $\mathbf{w}_i^T$ of T-RBMs with $n_h = 400$ for the $2d$ Ising model at temperature $T = 2.25$. Mean of standard deviation $\langle std_{T=2.25}\rangle$ of $\mathbf{w}_i^T$ is calculated. The colorbar range is set within $[-3 \times \langle std_{T=2.25}\rangle, 3 \times \langle std_{T=2.25}\rangle]$.

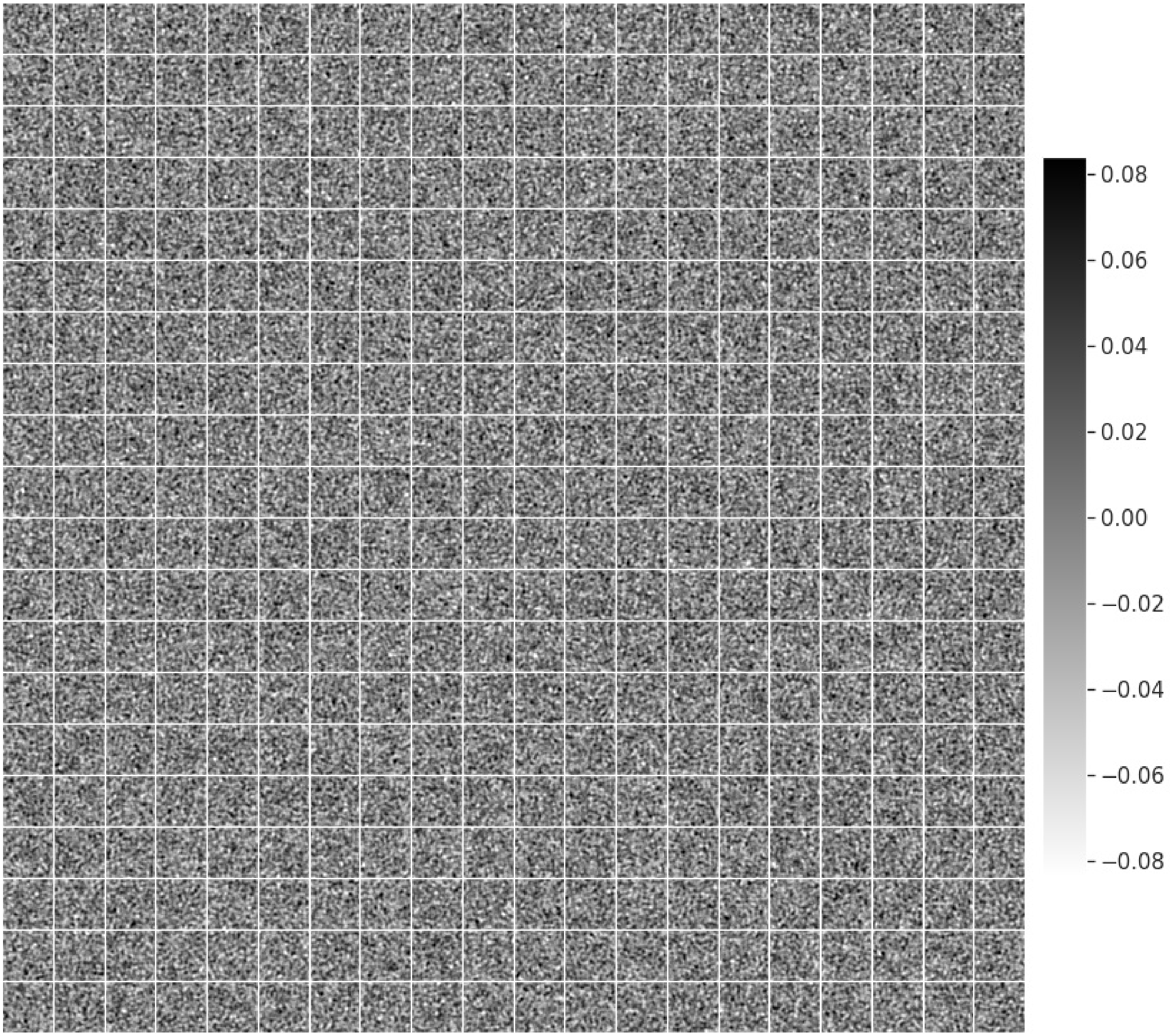

Figure S7. Filters $\mathbf{w}_i^T$ of T-RBMs with $n_h = 400$ for the $2d$ Ising model at temperature $T = 3.5$. Mean of standard deviation $\langle std_{T=3.5}\rangle$ of $\mathbf{w}_i^T$ is calculated. The colorbar range is set within $[-3 \times \langle std_{T=3.5}\rangle, 3 \times \langle std_{T=3.5}\rangle]$.

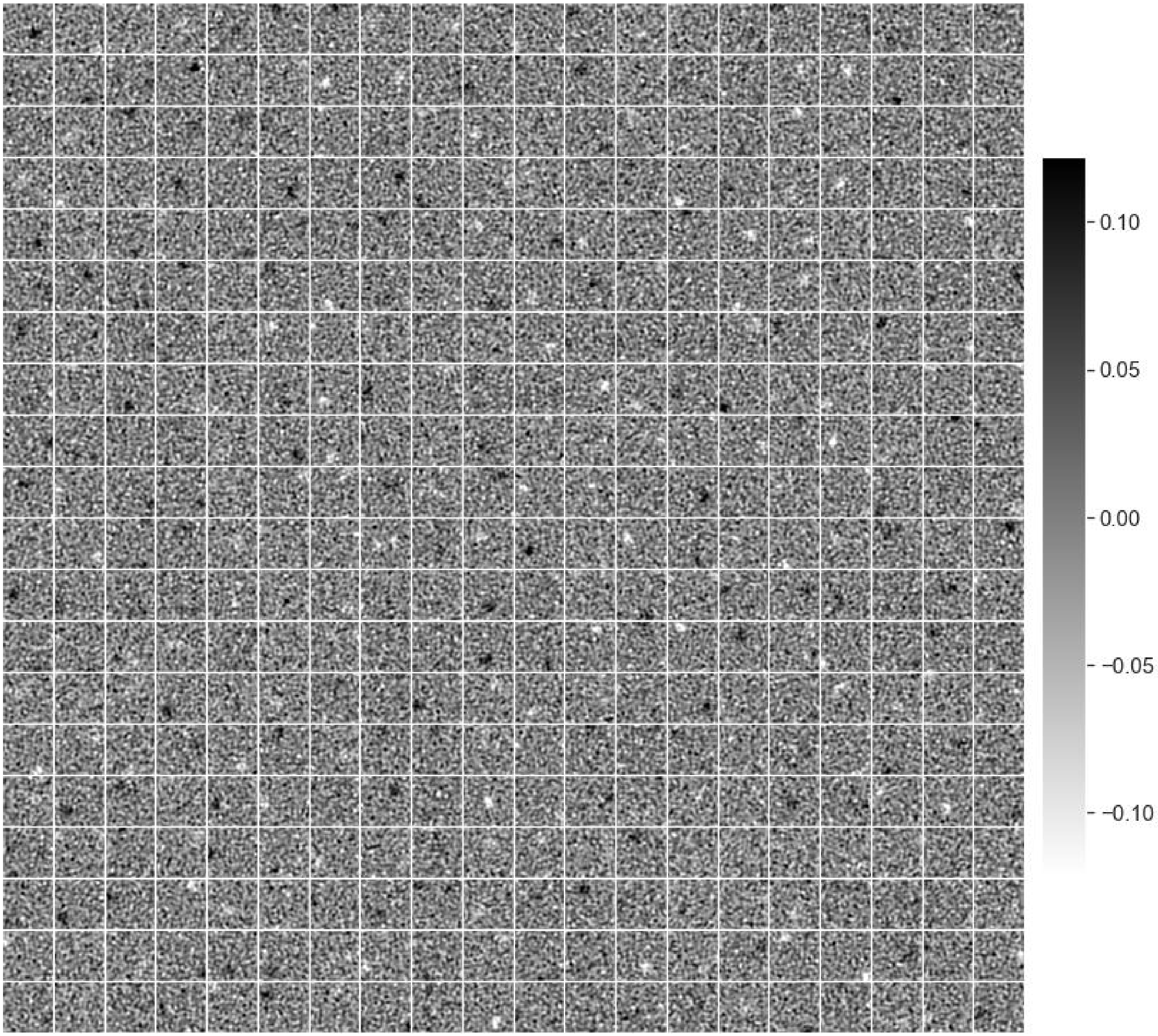

Figure S8. Filters $\mathbf{w}_i^T$ of $\cup T$-RBMs with $n_h = 400$ for the $2d$ Ising model. Mean of standard deviation $\langle std \rangle$ of $\mathbf{w}_i^T$ is calculated. The colorbar range is set within $[-3 \times \langle std \rangle, 3 \times \langle std \rangle]$.

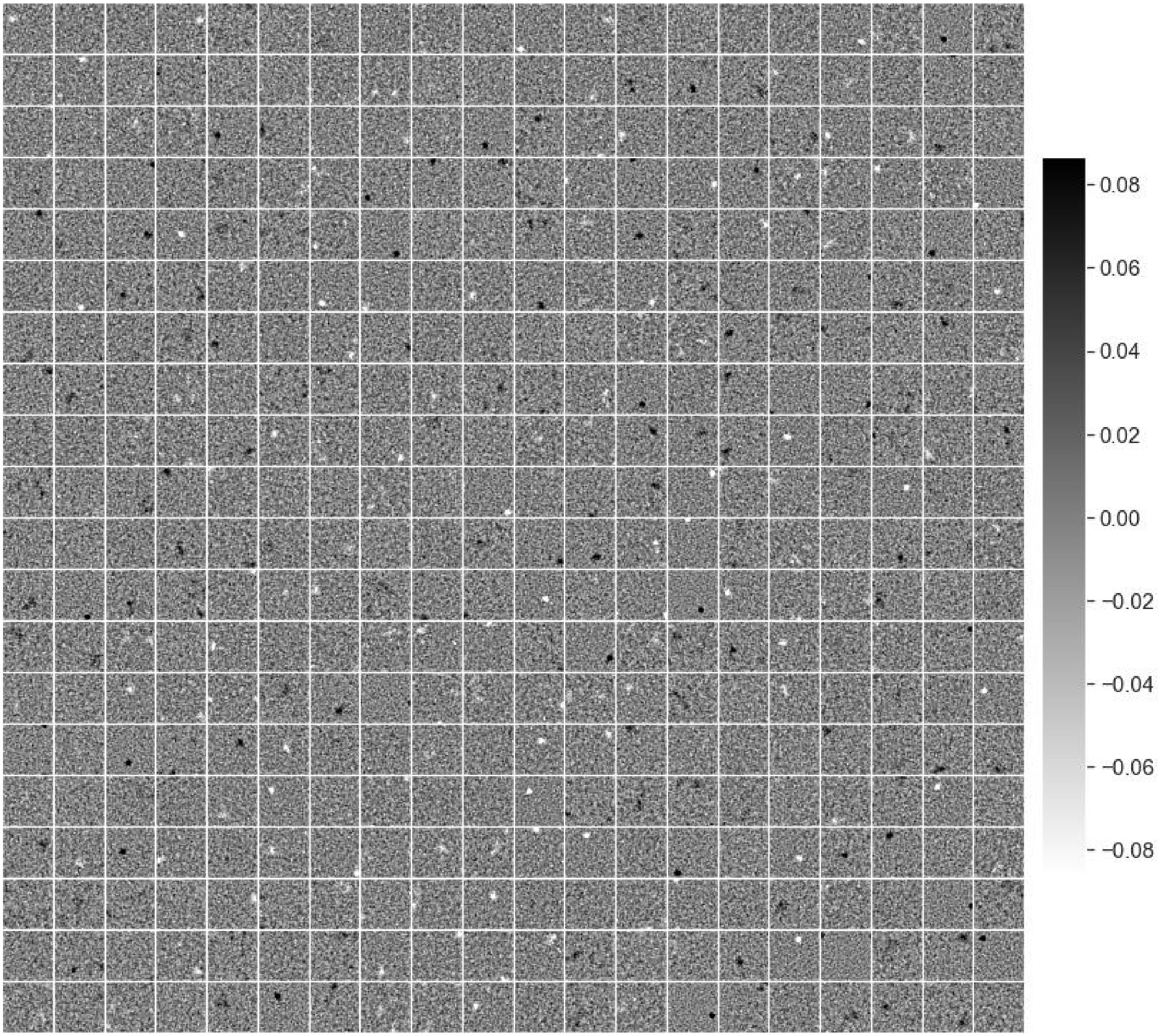

Figure S9. Filters $\mathbf{w}_i^T$ of $\cup T$-RBMs with $n_h = 1600$ for the $2d$ Ising model. Mean of standard deviation $\langle std \rangle$ of $\mathbf{w}_i^T$ is calculated. The colorbar range is set within $[-3 \times \langle std \rangle, 3 \times \langle std \rangle]$.
